# The Coevolution of Virulence: Tolerance in Perspective

**DOI:** 10.1371/journal.ppat.1001006

**Published:** 2010-09-09

**Authors:** Tom J. Little, David M. Shuker, Nick Colegrave, Troy Day, Andrea L. Graham

**Affiliations:** 1 Institute of Evolutionary Biology, Centre for Immunity, Infection and Evolution, School of Biological Sciences, University of Edinburgh, Edinburgh, United Kingdom; 2 School of Biology, University of St Andrews, St Andrews, Fife, United Kingdom; 3 Departments of Mathematics & Biology, Queen's University, Kingston, Ontario, Canada; 4 Department of Ecology & Evolutionary Biology, Princeton University, Princeton, New Jersey, United States of America; University of California San Diego, United States of America

## Abstract

Coevolutionary interactions, such as those between host and parasite, predator and prey, or plant and pollinator, evolve subject to the genes of both interactors. It is clear, for example, that the evolution of pollination strategies can only be understood with knowledge of both the pollinator and the pollinated. Studies of the evolution of virulence, the reduction in host fitness due to infection, have nonetheless tended to focus on parasite evolution. Host-centric approaches have also been proposed—for example, under the rubric of “tolerance”, the ability of hosts to minimize virulence without necessarily minimizing parasite density. Within the tolerance framework, however, there is room for more comprehensive measures of host fitness traits, and for fuller consideration of the consequences of coevolution. For example, the evolution of tolerance can result in changed selection on parasite populations, which should provoke parasite evolution despite the fact that tolerance is not directly antagonistic to parasite fitness. As a result, consideration of the potential for parasite counter-adaptation to host tolerance—whether evolved or medially manipulated—is essential to the emergence of a cohesive theory of biotic partnerships and robust disease control strategies.

## Introduction: What Controls Virulence?

Evolutionary biologists define the virulence of a parasite as the reduction in host fitness caused by infection. When this reduction in host fitness is due to an increased mortality rate, the consequences for parasite evolution are clear; host death means parasite death [1]. However, virulence can also include reductions in host fecundity [2], [3], which is relevant for some parasites such as castrating parasites and obligate killers [4], [5]. In either case, typically, virulence is reasoned to increase with an increasing parasite burden. Thus, conceptually, virulence may be viewed as resulting from the density of parasites within a host (*I*) and the degree of damage caused by each parasite (α, the per-parasite virulence):
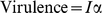
(1)This simple equation offers considerable insight into parasite evolution. For instance, it is generally assumed that parasites can achieve higher transmission success the more numerous they are within a host (higher within-host density), but that this will in turn lead to higher virulence, which may kill hosts more rapidly and compromise transmission success [6]. The resulting idea is that the most successful parasites cause an intermediate level of virulence, which encapsulates the trade-off model of virulence evolution (reviewed in [7]).

However, virulence is ultimately a pathology of the host [8], and thus will be jointly determined by both the parasite and host. The density of parasites within hosts, *I*, is controlled both by intrinsic replication rates of parasites and by the rate at which hosts kill parasites (e.g., [9]), while per-parasite virulence, α, can be controlled by parasite “virulence factors” such as toxins as well as host anti-toxin molecules. Some theoretical work has acknowledged this joint control of virulence—for example, by examining how host control of *I* via recovery rate affects coevolution [10], [11], or how host control of α via avoidance of immunopathology can shape parasite evolution [12]. Such studies, however, are in the minority, and the virulence literature has focussed primarily on parasite-controlled traits, despite the fact that virulence is of clear relevance to host evolution.

When thinking about host evolution, it can be helpful to discuss host fitness directly, rather than virulence. A simple linear model of host fitness is:
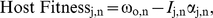
(2)which is host fitness in the absence of infection (ω_o,n_, the y-intercept) minus the terms that comprise virulence. Here *n* is the nth host genotype, and *j* is the jth parasite genotype. Many approaches to the study of host–parasite interactions have been less general than this. For example, much of the work on virulence has assumed constant α, or that *I* depends only on parasite genotype (e.g., [7]); work on host genetic control of resistance has often assumed that α is constant, but allowed *I* to depend on both parasite and host genotype (e.g., [13]); finally, work on the parameter α_n_ (often referred to as tolerance) tends to assume that it is a function only of host genotype (or that ω_o,n_ is constant [e.g., [14]]). While we appreciate that not every study needs to be holistic or coevolutionary, much might also be learned from a synthetic approach (see also [10], [15]).

In the following sections we emphasise why a full picture of the causes and consequences of virulence requires consideration of all components of Equation 2. We draw attention to the term ω_o,n_, which is not directly influenced by the parasite, and our discussion is weighted towards host genetic variation for tolerance (i.e., α_n_), both in terms of measurement and the evolutionary inferences that are possible. Despite these leanings, we aim to reinforce the notion that coevolutionary outcomes depend upon contributions from both interactors. Host-centric and parasite-centric views of virulence evolution have often been disconnected in the study of disease evolution, which has provided too narrow a perspective on the potential for interactions to generate dynamic coevolution. For example, the evolution of tolerance (i.e., selection on α_n_) has been thought of as having the potential to dampen coevolutionary dynamics, but this has rarely been with a full consideration of all the conditions that favour intense host or parasite counter-adaptation. Ultimately, host–parasite coevolution is about two interacting organisms gaining fitness at each other's expense, and a more holistic approach could unite a large range of perspectives on the evolutionary ecology of attack, defence, and commensalism.

## Tolerance and Intercepts

**Figure 1:**
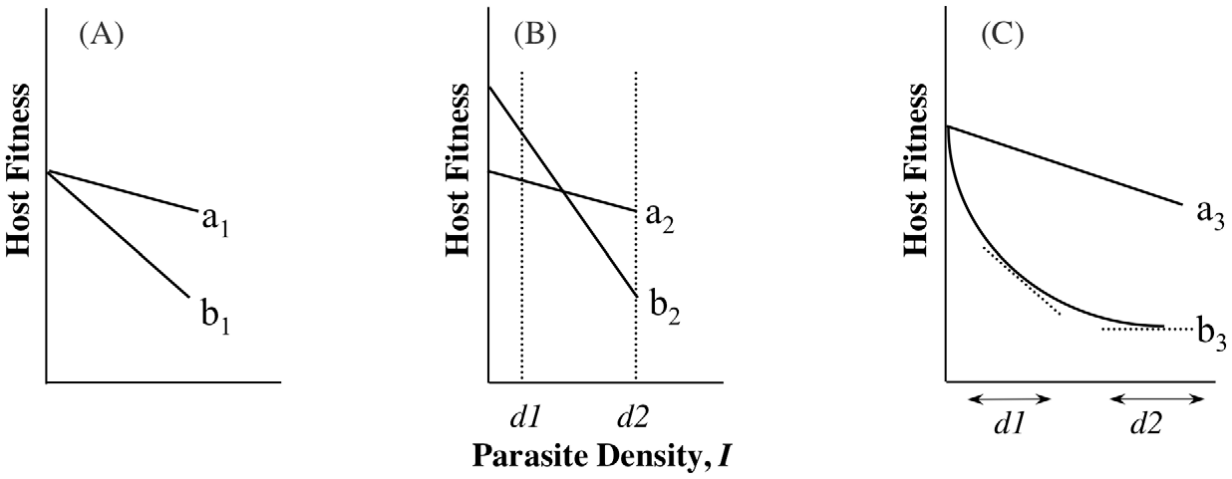
The importance of intercepts: point versus range tolerance. To understand differing interpretations of the evolutionary consequences of tolerance, it is necessary to consider when point and range tolerance will disagree. Below is one scenario where they will agree, and two where they may not. (A) With fitness in the absence of infection identical, at whichever parasite density measured, the fitness of the genotype with the flatter slope will be higher; here, genotype a_1_ is more tolerant than b_1_ regardless of how it is assessed. Both point and range tolerance measures therefore agree over the more tolerant genotype. (B) Here genotypes differ for their intercept, and the genotype with the higher point tolerance differs depending on whether parasite density is measured at *d1* (where b_2_>a_2_) or *d2* (where a_2_>b_2_). The fitness at *d1* is strongly influenced by fitness in the absence of infection, while fitness at *d2* is more strongly influenced by how fitness declines with increasing *I*. Under range tolerance, however, a_2_ is more tolerant, despite the fact that it is less (point) tolerant at low densities. (C) Here the point tolerance is always higher for a_3_, but the range tolerance depends upon the range of *I* considered; if tolerance is measured across the range depicted by *d1*, genotype b_3_ would be considered less tolerant, but it would be considered more tolerant if the range measured was *d2*. Genotype b_3_ is always less fit, however.

A recent experimental study of virulence in a rodent defined tolerance as the slope of the regression of host fitness on *I* [14]. This definition is similar to that from studies on plant responses to infection or herbivory (e.g., [16], [17]) and assumes that host differences in ω_o,n_ represent underlying differences in general vigour [14], [16], [17]. In addition to being measured as a slope, i.e., estimated across a range of parasite densities (range tolerance), tolerance has also been measured at one single parasite density (point tolerance) [18], [19], but depending on the relationship between α_j,n_ and ω_o,n_, the conclusions drawn from the different measures may not be the same (Box 1, Figure 1). There are yet further definitions of tolerance, and the possibility that different measures of tolerance will not always provide the same information presents challenges for reconciling theory with data and vice versa. A key step towards suitable comparison of the different tolerance measures will be to always measure host fitness when infected alongside fitness when uninfected, ω_o,n_ (Equation 2), although even this may be of limited value if per-parasite virulence (α) varies non-linearly with parasite density (*I*) (parasites get proportionally more or less benign with increasing density; Box 1, Figure 1C).

**Box 1. Density Ranges and Definitions of Tolerance**

The study of host-controlled α has been described as the study of tolerance: the ability of hosts to limit the damage caused by a given parasite burden, which is essentially the ability to minimize per-parasite virulence. It has been studied as a mean, i.e., where two genotypes carry the same parasite burden, but one genotype achieves higher fitness. We call this **point tolerance**. An empirical example of point tolerance is the striking genetic variation among strains of laboratory mice in the per-parasite virulence of *Streptococcus pneumoniae* infection [18]. Tolerance has also been studied as a slope to depict how quickly fitness falls as parasite density increases; more tolerant genotypes lose their fitness less quickly as densities increase (implying less sensitivity to changes in parasite numbers). We call this **range tolerance**. This has recently demonstrated for rodent malaria [14], using an approach in line with studies of tolerance to herbivory [16], [17].

Schematic examples of range and point tolerance are presented in Figure 1. The differences in interpretation implicit in these scenarios are important, as they would lead to different predicted evolutionary or epidemiological outcomes. These examples are meant to merely illustrate the potential confusion that may arise depending on how tolerance is measured, and we emphasise that this is not just a quibble about definitions. If we are to draw general conclusions about tolerance evolution, we need to resolve when empirical studies of point tolerance (e.g., [19]) should be freely compared with studies of range tolerance (e.g., [14]), and when either can inform theory that uses yet other definitions (e.g., [33]; see also [21]).

**Figure 2:**
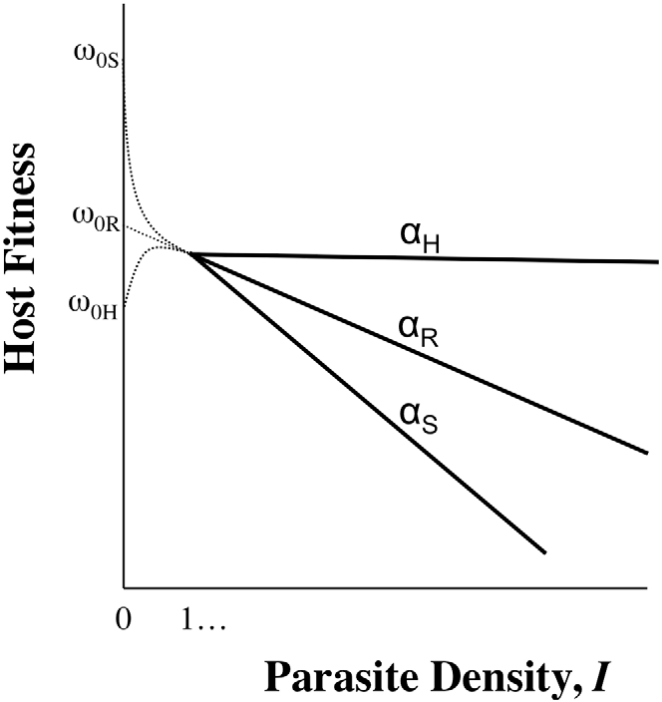
The importance of intercepts: pleiotropy. Host genotypes will almost certainly show differences in ω_o,n_ (genetic variation for life history characteristics is ubiquitous [37]), and in some cases these differences will be linked to variation in the traits that contribute to virulence (α_n_ or *I*_n_) via pleiotropy (where one gene influences more than one trait). For example, hosts that possess alleles that confer more potent defences (ability to control *I* or α) may be less fit when parasites are not around because the allele that aids defence compromises the performance of other traits (compare ω_oR_ and ω_oS_; R denotes resistance, S denotes susceptible). In other words, there may be a cost of possessing a defence mechanism [38], often referred to as a trade-off. It is even conceivable that ω_o,n_ is lower than host fitness at low *I*, because individuals without enough parasites can experience difficulty with immune regulation: the hygiene hypothesis posits that allergy and autoimmunity result from immune systems lacking direction from parasites ([39]; see ω_oH_, which denotes hygiene). Thus, the rank order of ω_o,n_ may be the opposite of the rank order of fitness when infected. Moreover, ω_on_ may not be easily predicted from the relationship between parasite density and host fitness when infected—for example, when just a small number of parasites stimulates a damaging or energy-sapping immune response that is little amplified by further infection. Generally, the fitness of uninfected individuals need not be a linear extrapolation of the relationship between fitness and parasite density (*I*).

Tolerance, by definition, does not include ω_o_, but inference about host evolution will be restricted when ω_o_ is not considered. First, there may be a biological relationship between tolerance and ω_o_ due to the effects of pleiotropy, in particular if defence is traded-off with other fitness traits (Figure 2). In this case, we could not disregard variation in ω_o,n_ as variation in general vigour that is independent of parasite-mediated selection. Moreover, variation in ω_o,n_, whether or not pleiotropy plays a role, will be crucial for determining evolutionary trajectories. Consider two host genotypes that differ for α but have equivalent ω_o_, so that one genotype has higher fitness across all values of *I* (compare genotypes in Figure 1A or 1C). In this case, the evolutionary outcome for hosts is certain, and α only determines the rate at which one host genotype replaces another. The more plausible and interesting scenario is where host genotypes differ for both α and ω_o_, but no genotype is the fittest across all parasite densities (Figure 1B). Relationships such as these can lead to the maintenance of host polymorphism, because the superiority of a genotype is entirely context dependent. Selection on tolerance traits can also maintain polymorphism when hosts recoup fitness in terms of fecundity instead of mortality [2]. In general, accurate estimates of evolutionary outcomes require that the slope of the parasitic relationship α (per-parasite virulence, or tolerance) be considered alongside the intercept ω_o_.

## The Problem of Intimacy

As with any symbiosis, when measuring genetic variation and inferring selection on disease-related traits, it is impossible to escape the issue that infection phenotypes represent the dual contribution of host and parasite. For example, when parasite burdens are measured in the field or as an experimental *outcome* (as opposed to an experimentally controlled variable), how can we know if parasite burden determines host fitness, or if host fitness, itself affected by a variety of environmental factors, is mediating parasite burden? Stressed hosts, for example, may express sensitivities that lead to differences in parasite load. Here, we would be examining genetic variation in laboratory-mediated stress; it would not be clear what defence trait would be selected upon and what evolutionary response we should expect to see. In this sense, the parasitic relationship is more comparable to correlated traits than to a norm of reaction.

The difficulty of using correlations to elucidate the trait under selection is evident when trying to envision the selection process that has shaped α_n_ and ω_o,n_ in a population. For example, host genotypes might differ in the fat reserves that are mobilized only in periods of stress (e.g., food shortage, temperature stress, and, of course, infection). If the effect of differences in fat reserves happen to scale with stress level and stress escalates with parasite density, then hosts would differ in range tolerance. Whilst this would be tolerance of infection in a very broad sense, it might have evolved for reasons independent of infection. Conversely, differences in general vigour might arise via parasite-mediated selection. For example, a molecule that mops up pathogen-produced toxins without depressing pathogen numbers (clearly a tolerance mechanism that could affect the slope, α), might later be recruited to mop up free radicals produced during respiration in the absence of infection (raising ω_o_). These simple hypothetical examples reinforce the problem of separating slopes from intercepts when inferring selection: variation in ω_o,n_ can appear as just variation in general vigour, and not subject to parasite-mediated selection (when in fact a process of parasite-mediated selection has modified ω_o_), whilst slopes may appear to be subject to parasite-mediated selection (when in fact slope differences arose through selection on ω_o_).

It is thus difficult to infer an underlying mechanism from correlational estimates of tolerance, and it is likely to be important to delve into mechanistic studies (e.g., [19]). For many, the terms “resistance” and “tolerance” carry with them some connotation of mechanism (e.g., [20], [21]), and, just as different mechanisms of resistance (e.g., when a lack of infection comes about via behavioural avoidance versus an immune reaction [22]) should predict very different trajectories of selection, different mechanisms of tolerance also predict different evolutionary trajectories. Knowing the mechanisms that confer tolerance or resistance will shed light on host evolution, and ultimately coevolution. Generally, it will be interesting to understand why the relationship between *I* and host fitness might become steeper or change shape: is it due to an immune system molecule that blocks toxins, for example, or prevents immunopathology [23], but only over certain parasite density ranges? Or is it simply that the environment and subsequent condition of the host mediate a change in the severity of parasitism? Indeed, it is easy to envision an additional factor added to Equation 2: the subscript *e* denoting the effects of the environment on the parasitic relationship. The effects of factors such as host density, resource availability, and temperature are obvious avenues of study here [24]–[26].

This problem of intimacy will often extend to the study of genetic variation: *I*_j,n_, and α_j,n_ are the product of two interacting genomes (Equation 2), and it is often difficult to identify which antagonist is controlling the infection phenotype due to genotype-by-genotype interactions. For example, the defence capabilities of a host genotype often depend on which parasite genotype is involved, while at the same time the impact of a particular parasite genotype depends on the host genotype it infects [9], [15], [27]–[30]. Nonetheless, previous definitions of tolerance have implicitly assumed that the sensitivity of host fitness to parasite burden (i.e., α) is entirely determined by host genotype [14]. But this sensitivity could plausibly depend on an interaction between the host and parasite genotypes as well, further calling into question simplified measures of tolerance.

## Coevolution

Far from just presenting challenges for the study of parasitism, genotype-by-genotype interactions actually represent the foundation of most host–parasite coevolutionary models [9], [27], [28]. Usually this is studied in the context of resistance to infection, where the relative performance of different parasite genotypes (in terms of *I*_j,n_) depends on which host genotype they infect. If *I*_j,n_ and thereby virulence are determined by a host–parasite genetic interaction, then this can lead to antagonistic coevolutionary dynamics and provide a mechanism for the maintenance of genetic polymorphism (specifically, negative frequency-dependent selection [e.g., [31]]). Above and in Figure 1, we highlighted how some scenarios of variation in α and ω_o_ should also promote host polymorphism (see also [2]), and this can be extended to a full treatment of host-parasite coevolution. Here, coevolutionary dynamics will be driven by the rules of virulence optimisation. Selection for tolerance will change the selection gradient on parasites, and present them with a new optimum (or adaptive peak), towards which they will evolve. It has been suggested [14] that the evolution of tolerance dampens coevolution by not directly reducing parasite numbers, but we posit that any host evolution that knocks parasites away from their evolutionary optimum (or creates a new one) will be countered by parasite evolution.

We do not yet have a clear view on the nature of the dynamics generated by parasite evolution in response to new adaptive peaks that arise due to tolerance evolution. The tolerance theory thus far largely omits host (e.g., [32]) or parasite (e.g., [33]) counter-adaptations. Where coevolution has been permitted in optimality models, however, it has been clearly shown that parasite evolution is highly sensitive to host evolution [34]. Further investigation of host–parasite coevolution that allows host genotypes to exhibit heterogeneity in tolerance is needed, for both theoretical and practical reasons.

For instance, we do know that one step of host evolution towards greater tolerance can select for parasites with higher replication rates and populations that suffer a greater parasite burden [32]. Thus, one plausible coevolutionary scenario is that hosts that evolve ever greater tolerance can select for the evolution of parasites with higher growth rates and increased transmission [32] because the cost of virulence, in terms of killing the host prior to transmission, will be reduced. In this way, the evolution of tolerance mirrors attempts made by medical interventions (particularly vaccination) to ameliorate the pathology associated with infection, without necessarily eradicating the infection or reducing parasite densities. Such vaccination strategies can select for more virulent pathogens, presenting grave risks for those who come into contact with the more virulent parasite but are not vaccinated [35], [36]. The same is true for the spread of tolerance through populations: tolerant individuals may allow parasites to evolve greater virulence, likewise causing grave risks for intolerant or migrant individuals that become exposed to the disease [32]. Such an outcome could be important for human disease risk.

Thus, although local tolerance evolution can even lead to commensalisms [32], [33], when it is coupled with geographic structuring of populations or infrequent contact between species, tolerance evolution in one population might underlie why some zoonoses or other emerging diseases are particularly devastating to other populations. Indeed, apparent paradoxes such as “tolerance evolution might be bad” represent key lessons from viewing disease in a coevolutionary context. The risk that tolerance evolution could increase disease severity in intolerant hosts may be just scratching the surface. Equation 2 suggests a set of critical components that can be compared across host and parasite genotypes (or environments) to gain insight into host evolution, parasite evolution, or coevolution. We encourage use of such a unified, coevolutionary framework, rather than host-centric or parasite-centric alternatives, to achieve a true understanding of tolerance and to shed light on disease control strategies that will not provoke undesirable pathogen evolution. Ultimately, unification is essential if we are ever to achieve a universal theory of disease severity, or indeed a universal theory of biotic partnerships.
